# The Central Sensitization Inventory Measures Thoughts and Emotions

**DOI:** 10.1177/23743735241273589

**Published:** 2024-08-14

**Authors:** Sina Ramtin, Marielle Ngoue, David Ring, Teun Teunis

**Affiliations:** 1 Department of Surgery and Perioperative Care, Dell Medical School, University of Texas at Austin, Austin, TX, USA; 2 Department of Orthopaedic Surgery, University Pittsburgh Medical Center, University of Pittsburgh, Pittsburgh, PA, USA

**Keywords:** Central Sensitization Inventory, thoughts, emotions, anxiety, mental health, cross-sectional, factor analysis

## Abstract

To determine if the Central Sensitization Inventory questionnaire (CSI) functions as a mental health measure among a cross-section of people seeking musculoskeletal specialty care, we asked: (1) What is the association of CSI total score and item groupings identified in factor analysis with mental health measures? and (2) What is the association between specific CSI items that represent each factor well and specific mental health measures? One hundred and fifty-seven adults seeking specialty care for musculoskeletal symptoms completed the CSI, a measure of catastrophic thinking, and 3 measures of distress (symptoms of health anxiety, general anxiety, and depression). Exploratory factor analysis was used to identify item groupings. Exploratory factor analysis identified 4 item groupings (factors): (1) thoughts and feelings (mental health), accounting for 52% of the variation in the CSI, (2) urinary and visual symptoms (15%) (3) body aches (10%), and (4) jaw pain (8.1%). More than half the variation in both the CSI total score (51%) and the thoughts and feelings factor (57%) were accounted for by variation in measures of catastrophic thinking and distress. Specific items that account for large amounts of the variation in the CSI also had notable correlations with mental health measures. The strong relationship between the CSI and thoughts and emotions suggests that the CSI functions largely as a mental health measure. If the concept of central sensitization is to help people get and stay healthy, it will depend on evidence that central sensitization can be measured and quantified distinct from mental health.

## Introduction

### Background

The concept of Central Sensitization (CS)—a term coined in 1983—proposes that illnesses characterized by persistent, diffuse pain without limited measurable peripheral pathophysiology are the result of central nervous system pathophysiology.^1^ This spectrum of illnesses is addressed in mental health terms as functional somatic syndromes.^2^ Specific examples of this class of human illness may include fibromyalgia, irritable bowel syndrome, temporomandibular disorder, and interstitial cystitis, among others.^3^ It is possible that CS and functional somatic syndromes (or somatic symptom disorders) are, respectively, alternative biomedical and biopsychosocial conceptualizations of pain as an illness.

### Rationale

It is not clear that there is a distinction or an advantage to speaking of brain (neurons and neurotransmitters) rather than mind (thoughts and emotions). There are several drawbacks of focusing on pathophysiology rather than mental health. Foremost is the potential reinforcement of mental health stigma. As if it is better to have a nerve disease than to feel hopeless or experience distorted thinking. Second is the concept of CS may emphasize external pharmaceutical treatments, which might reinforce passivity and magical thinking (limited health agency) and distract people from effective mindset training such as cognitive behavioral therapy. Cultivating healthy mindsets alleviates symptoms from health anxiety/functional somatic syndromes^4,5^ and improves comfort and capability for discrete pathologies.^5,6^ This is true even if we eventually discover an important central neuropathophysiology.

The concept of CS only has value to the extent that we can objectively, experimentally, reliably measure aspects of pathophysiology that are beyond a person's control and cannot be modified by efforts to improve mindset and circumstances.

In the absence of a method to objectively verify or falsify the hypothesis of overactive neural pathways in the central nervous system in individual patients, the concept of “central sensitization” is measured using the Central Sensitization Inventory (CSI).^7–16^ If it can be confirmed that the CSI measures mental health, then diagnosis of CS will need to move away from questionnaire-based diagnosis and instead measure the theorized neuropathophysiology directly.

### Study Questions

To determine if variation in the CSI is associated with variations in mindsets (in other words, if the CSI functions as a mental health measure), we asked: (1) What is the association of CSI total score, and item groupings identified in exploratory factor analysis, with mental health measures? (2) What is the association between individual CSI items that represent the identified factors and specific mental health measures?

## Methods

### Study Design and Setting

This was a cross-sectional study of people seeking specialty care for an upper extremity, lower extremity, or spine musculoskeletal condition in one of four urban offices in the United States.

### Participants

Between October 2021 and November 2021, 157 new and returning, English-speaking adults (over 18) agreed to participate. The questionnaires were administered on the internet-based Health Insurance and Portability Accounting Act-approved Research Electronic Data Capture system at the end of their visit with musculoskeletal specialists. Inclusion criteria were English fluency and literacy. Exclusion criteria were English illiteracy and cognitive dysfunction precluding completion of questionnaires. This study was approved by the institutional review board. Fifty-three patients were women (83%), and the mean age was 51 (standard deviation, 18) years (Table 1).

**Table 1.** Patient Demographics.^a^

Variable	Value
N	157
Age	51 ± 18
Gender	
Man	47% (73)
Woman	53% (83)
Nonbinary	1% (1)
Race	
White	64% (101)
Nonwhite races (eg, African-American, Asian, etc)	36% (56)
Marital status	
Married or partnered	55% (87)
Single/Divorced/Separated	45% (70)
Education	
High school	18% (28)
2-year college	13% (21)
4-year college	43% (68)
degree (eg PhD)	25% (40)
Work status	
Employed	64% (100)
Unemployed/ Other	36% (57)
Household income	
<$24,000	10% (15)
$24,000-$46,000	10% (15)
$46,000-$75,000	21% (33)
$75,000-$121,000	26% (41)
>$121,000	34% (53)
Insurance status	
Medicare	29% (45)
Private	57% (89)
Other	15% (23)
Continuous variables	
Age	52.3 ± 17.8
CSI	18.6 ± 9.8
SHAI-5	3.6 ± 2.1
PHQ-2	0.8 ± 1.1
GAD-2	1.1 ± 1.4
PCS-4	2.9 ± 3.3

Abbreviations: CSI, Central Sensitization Inventory; SHAI-5, The Short Health Anxiety Inventory; PHQ-2, Patient Health Questionnaire; GAD-2, general anxiety disorder; PCS-4, Pain Catastrophizing Scale.

^a^ Continuous variables as mean ± standard deviation; categorical variables as percentage (number).

### Questionnaires of CS and Mental Health

We quantified the construct of CS using part A of the CSI.^17^ Part A of the CSI consists of 25 items that address CS symptoms rated on a 5-point Likert scale from 0 (never) to 4 (always), with a total possible score of 100. A higher score indicates greater CS. Part B of the CSI addresses prior diagnosis of generalized anxiety disorder, restless leg syndrome, or fibromyalgia, and was excluded.^18^ Each questionnaire was administered to the patient on a single page. The sequence of the questionnaire was not randomized, as a prior study revealed that no significant differences existed in the mean, median, and internal consistency of intermixed and fixed-order items.^19^

Health anxiety was measured using the five item Short Health Anxiety Inventory (SHAI-5). Total scores range from 0 to 15 with a higher score indicating greater health anxiety.

Symptoms of depression were measured using the 2-item version of the Patient Health Questionnaire (PHQ-2). Items address how often participants are bothered by “little interest or pleasure in doing things” and “feeling down, depressed or hopeless.” Each answer is rated on a 4-point Likert scale ranging from 0 “not at all” to 3 “nearly every day.” A higher score indicated more symptoms of depression.

Symptoms of anxiety were measured using the 2 item Generalized Anxiety Disorder questionnaire (GAD-2). Items include “feeling nervous, anxious, or on edge” and “not being able to stop or control worrying,” and answers range from 0 “not at all” to 3 “nearly every day.” Total score ranges from 0 to 6 with a higher score indicating greater anxiety symptoms.

We measured unhelpful thoughts and feelings of distress about symptoms using the 4-item version of the Pain Catastrophizing Scale (PCS-4). Items include “I worry all the time about whether the pain will end” and “I anxiously want the pain to go away,” and answers range from 0 “not at all” to 4 “all the time.” Total scores range from 0 to 16 with a higher score indicating worse catastrophic thinking.

### Other Variables

Demographic information included age, gender, income level, employment status, education level, and marital status.

### Primary and Secondary Study Outcomes

Our study goal was to determine the degree to which variation in CS is associated with variation in thoughts and emotions (mental health). We therefore first sought to determine the association of the CSI total score, and groups of items within the CSI, with mental health measures, using Spearman rank correlation. We measured 95% confidence intervals through bootstrapping (n = 1000). We also determined how much of the variation in CSI and the underlying item groupings is explained by mental health by including all mental health measures as independent variables in a multivariable linear regression model.

Secondly, we determined the association of each mental health measure with individual items that best represent the CSI item grouping through spearman rank correlation with bootstrap 95% confidence intervals.

### Statistical Analysis

An a priori power analysis indicated that 124 participants would provide 0.80 power to detect a correlation of 0.25, with alpha set at .05. This means that a mental health measure would account for about 6% of the variation in the groups of items identified within the CSI. Based on previous experience, we increased our sample size to 157 participants to be able to perform a reliable factor analysis. In addition, a previous simulation study indicated that good reliability (assuming a reliability criterion 0.92, a variable to factor ratio of 5:1, and a 4 factor solution) can be achieved with 110 participants assuming wide communality (factor loading between 0.2 and 0.8).^20^

To determine to what extent CSI items measure specific underlying constructs (or factors), we performed an exploratory factor analysis. We used an eigenvalue (a measure of the amount of variance in the data accounted for by a set of items) greater than 1.0 along with the elbow method on the graphic representation of eigenvalues to select the number of item groupings. We performed a confirmatory factor analysis to identify the individual items that best represent each group of items within the CSI. We included items with a coefficient greater than 0.60 on confirmatory factor analysis. This means that if an item grouping increases 1 point in variance, the item has to at least increase 0.60 point.

## Results

### Exploratory and Confirmatory Factor Analysis

On exploratory factor analysis we identified 4 item groupings (Appendix 1). The scree plot levels off after 3 groups of items, at an eigenvalue of 1.08 (Appendix 2). However, 3 groups of items, accounting for 77% of the variation in CSI, left several head and neck items unrepresented. We therefore decided on 4 groups of items: (1) thoughts and feelings (mental health), accounting for 52% of the variation in the CSI, (2) urinary and visual symptoms, accounting for 15% (3) body aches, accounting for 10%, and (4) jaw pain, accounting for 8.1% (Table 2). In confirmatory factor analysis, there were 5 items that represented the thoughts and feelings (mental health) item group; 2 items that represented body aches symptoms, and 1 item each representing the urinary/visual symptoms and jaw pain groups (Table 3). The measures of model fit indicate the exploratory factor model fits the data well (Appendix 3). This indicates that if we would repeat the study in a similar population, we would likely find similar results.

**Table 2.** Correlation of Central Sensitization Question Groupings With Mental Health Measures.^a^

	SHAI-5 (health anxiety)			PHQ-2 (symptoms of depression)			GAD-2 (symptoms of anxiety)			PCS-4 (distress about symptoms)			
	Spearman correlation coefficient (95% CI)	SE	*P* value	Spearman correlation coefficient (95% CI)	SE	*P* value	Spearman correlation coefficient (95% CI)	SE	*P* value	Spearman correlation coefficient (95% CI)	SE	*P* value	Variation explained (R2)
Complete Central Sensitization Inventory	0.55 (0.42-0.67)	0.06	**<**.**001**	0.42 (0.28-0.56)	0.07	**<**.**001**	0.47 (0.33-0.60)	0.07	**<**.**001**	0.43 (0.29-0.57)	0.07	**<**.**001**	**0**.**51**
Thoughts and feelings	0.51 (0.39-0.64)	0.06	**<**.**001**	0.56 (0.44-0.69)	0.06	**<**.**001**	0.57 (0.44-0.69)	0.06	**<**.**001**	0.32 (0.17-0.48)	0.08	**<**.**001**	**0**.**57**
Urinary and visual symptoms	0.38 (0.22-0.53)	0.08	**<**.**001**	0.29 (0.13-0.45)	0.08	**<**.**001**	0.36 (0.22-0.50)	0.07	**<**.**001**	0.23 (0.08-0.39)	0.08	.**004**	**0**.**22**
Body aches	0.40 (0.26-0.55)	0.07	**<**.**001**	0.24 (0.07-0.40)	0.08	.**005**	0.24 (0.08-0.39)	0.08	.**003**	0.48 (0.34-0.61)	0.07	**<**.**001**	**0**.**37**
Jaw pain	0.34 (0.18-0.49)	0.08	**<**.**001**	0.23 (0.07-0.39)	0.08	.**004**	0.27 (0.12-0.42)	0.08	**<**.**001**	0.30 (0.15-0.45)	0.08	**<**.**001**	**0**.**20**

Abbreviations: SHAI-5, The Short Health Anxiety Inventory; PHQ-2, Patient Health Questionnaire; GAD-2, General Anxiety Disorder; PCS-4, Pain Catastrophizing Scale; SE, standard error.

^a^ We used the adjusted R^2^, this measures the variation in each question grouping accounted for by all mental health measures. All correlations are Spearman rank with nonparametric bootstrap (n = 1000) for 95% interval.

**Table 3.** Best Questions Measuring Aspects of the Central Sensitization Questionnaire.^a^

Question groupings	Question	Coefficient	Standard error	Variation explained	SHAI-5 (health anxiety)	PHQ-2 (symptoms of depression)	GAD-2 (symptoms of anxiety)	PCS-4 (distress about symptoms)
					*Spearman correlation with 95% CI*			
Thoughts and feelings	1. I feel tired and unrefreshed	0.80 (0.73-0.87)	0.037	0.63	0.45 (0.31-0.58)	0.53 (0.40-0.65)	0.44 (0.30-0.59)	0.34 (0.19-0.49)
	13. I have difficulty concentrating	0.64 (0.53-0.75)	0.055	0.41	0.28 (0.13-0.44)	0.32 (0.18-0.47)	0.38 (0.24-0.53)	0.08 (−0.08 to 0.25)
	15. Stress makes my physical symptoms get worse	0.61 (0.49-0.72)	0.057	0.37	0.43 (0.30-0.56)	0.38 (0.24-0.52)	0.37 (0.21-0.53)	0.33 (0.19-0.48)
	16. I feel sad or depressed	0.66 (0.55-0.76)	0.053	0.43	0.32 (0.17-0.47)	0.52 (0.40-0.63)	0.56 (0.45-0.67)	0.20 (0.03-0.37)
	17. I have low energy	0.82 (0.75-0.89)	0.036	0.67	0.46 (0.34-0.58)	0.49 (0.36-0.62)	0.42 (0.28-0.55)	0.32 (0.16-0.48)
Urinary and visual symptoms	7. I am sensitive to bright lights	0.66 (0.52-0.81)	0.074	0.44	0.22 (0.06-0.37)	0.19 (0.03-0.34)	0.22 (0.06-0.38)	0.17 (0.01-0.32)
Body aches	2. My muscles feel stiff and achy	0.71 (0.60-0.82)	0.057	0.50	0.36 (0.22-0.50)	0.14 (−0.02 to 0.30)	0.15 (−0.02 to 0.31)	0.34 (0.19-0.49)
	9. I feel pain all over my body	0.75 (0.64-0.85)	0.053	0.56	0.42 (0.30-0.54)	0.23 (0.07-0.39)	0.23 (0.08-0.39)	0.44 (0.30-0.58)
Jaw pain	18. I have muscle tension in my neck and shoulders	0.77 (0.61-0.94)	0.086	0.60	0.31 (0.17-0.45)	0.29 (0.13-0.45)	0.31 (0.16-0.46)	0.31 (0.16-0.47)

Abbreviations: SHAI-5, The Short Health Anxiety Inventory; PHQ-2, Patient Health Questionnaire; GAD-2, General Anxiety Disorder; PCS-4, Pain Catastrophizing Scale.

^a^ Included questions with a coefficient of 0.60 or greater on confirmatory factor analysis.

### Association of the CSI, and Groups of Items Within the CSI, With Mental Health Questionnaires

There was a strong correlation of the CSI total score with mental health measures from 0.42 (95% CI 0.28-0.56) for symptoms of depression (PHQ-2) to 0.55 (95% CI 0.42-0.67) for symptoms of health anxiety (SHAI-5). Collectively, variation in mental health measures together accounted for 51% of the variation in CSI. Correlation with the specific item groupings (factors) identified in exploratory factor analysis varied from modest for the relationship of head and neck symptoms factor with symptoms of depression (PHQ-2; r = 0.23 [95% CI 0.07-0.39]; 20% variation accounted for in multivariable models) to strong for the relationship of the unhelpful thoughts factor to symptoms of anxiety (GAD-2; r = 0.57 [95% CI 0.44-0.69]; 57% of the variation in multivariable models (Table 2).

### Association of Important CSI Items and Mental Health Measures

The smallest correlation with individual items was between the item “I have difficulty concentrating” and distress about symptoms (PCS-4; 0.08 [95% CI −0.08 to 0.25]). The largest correlation was between “I feel sad or depressed” and symptoms of depression (PHQ-2; 0.56 [95% CI 0.45-0.67]; (Table 3).

## Discussion

### Background and Rationale

The concept of CS posits altered central nervous system physiology. Proposed measures of CS include tests of physical pain thresholds and the CSI, a questionnaire with items that address mental health and a variety of physical symptoms. Prior investigations have demonstrated larger associations of the CSI with mental health measures than with physical pain threshold tests.^8,17^ If the CSI is largely measuring thoughts and feelings about symptoms, why not instead develop and promote improved mental health measures that feel comfortable to patients and result in useful discussions regarding the importance of attention to mental and social health along with treatment of physical symptoms? Consistent with the results from studies in other settings, our study of people seeking specialty care for musculoskeletal symptoms found that variation in the CSI associates largely with variation in mental health.

### Limitations

This study had a number of limitations. First, the patient population had limited gender (83% women) and language diversity, was mostly people with upper extremity musculoskeletal conditions and may not be representative of other settings. In our opinion, there is sufficient diversity in the responses that the associations are likely reproducible in other settings. It seems plausible that the findings would be similar among people with other types of pain. Second, we included both new and established patients. Prior studies found no association with visit type.^21,22^ Third, the large number of measures, many containing similar sounding items, can be associated with questionnaire fatigue, but this has not proved to be an issue with prior similar analyses. Fourth, there is some subjectivity in the determination of the number of factors and the concepts assigned to them. The group of mental health items are relatively unambiguous.

### Association of CSI, and Groups of Items Within the CSI, With Mental Health Questionnaires

The observation that variation in the CSI and its statistical item subgroupings is notably related to variations in mental health measures brings into question the ability of the CSI to measure pathophysiology distinct from mental health. Indeed, the other factors identified may represent an enumeration of somatic symptoms from various areas, which resemble a measure of somatic symptom disorder. There may be a need to choose whether we interpret CSI scores through a biomedical or biopsychosocial lens. The study documenting the development of the CSI evaluated 359 participants (140 from the general population and 210 with a chronic condition) and identified a similar 4-factor structure, but there were different relative variations in the CSI accounted for by the factors: (1) Physical symptoms (31%), (2) Emotional distress (7.2%), (3) Headache/jaw symptoms (10%), and (4) Urological symptoms (5.2%).^23^ In subsequent studies, variations in mental health accounted for much more of the variation in CSI scores, as observed in the current study. For instance, in a cross-sectional study of 114 patients seeking care for persistent pain there was a strong association of CSI with a comprehensive mental health measure and a moderate correlation with a measure of widespread pain designed for patients with fibromyalgia.^9^ In another study of 115 people in care for temporomandibular pain and 31 controls, scores on the CSI correlated with nonspecific physical symptoms (ρ = 0.68), symptoms of depression (ρ = 0.78), and symptoms of anxiety (ρ = 0.62).^8^ Mental health was also important in a cross-sectional secondary analysis of 78 people with unilateral shoulder pain of less than 6 months and current pain where CSI was associated with a measure of resilience, a measure of symptoms of anxiety, and a measure of negative affect.^24^ The strong relationship between the CSI and mental health measures seems consistent and notable.

### Association of Important CSI Items and Mental Health Questionnaires

The finding of notable correlations between the CSI items that best represent their item grouping (factor) with mental health measures further supports the notion that CSI is largely measuring mental health. Inadequate consideration of the overlap between concepts such as CSI and aspects of mental health has the potential of overemphasizing the biomedical paradigm, which is a strategy used—arguably inappropriately at times—to support pharmaceutical sales.^25,26^ Given the known relationship between unhealthy mindsets (misconceptions, worry, despair) and the diffusion and intensity of symptoms,^27–31^ it is not clear that items from symptom inventories and mental health measures can be repackaged into a measure meant to quantify or represent a speculative central neuropathology. And if the concept of CS is meant to facilitate biopsychosocial treatment strategies with less potential for patient offense in the face of mental health stigma, quantitative and qualitative studies comparing physiological and psychological explanations of disproportionate pain find that neither explanation avoids the stigma associated with mental health.^25,26^

## Conclusion

The findings of this and other studies confirm that the CSI is a measure of mental health and it may be inappropriate to use it to measure, quantify, or diagnose a unique neuropathophysiology. More importantly, the concept of CS and use of the CSI to diagnose it risks medicalizing mental health which can further reinforce mental health stigma and discourage people from working on their mindset and adopting a habit of mindset exercises. The pattern of patient somatization of distress, physician speculative pathophysiologies, and consequent stigmatization of mental health are intertwined, with many important manifestations over the last 200 years including hysteria, whiplash, and repetitive strain injury.^32^ In our opinion, if there is utility to the concept of CS for helping people get and stay healthy, it will depend on evidence that there is a pathophysiology (CS) that can be measured, quantified, and treated distinct from mental health.

## Supplemental Material

sj-docx-1-jpx-10.1177_23743735241273589 - Supplemental material for The Central Sensitization Inventory Measures Thoughts and Emotions Supplemental material, sj-docx-1-jpx-10.1177_23743735241273589 for The Central Sensitization Inventory Measures Thoughts and Emotions by Sina Ramtin, Marielle Ngoue, David Ring and Teun Teunis in Journal of Patient Experience

sj-docx-2-jpx-10.1177_23743735241273589 - Supplemental material for The Central Sensitization Inventory Measures Thoughts and Emotions Supplemental material, sj-docx-2-jpx-10.1177_23743735241273589 for The Central Sensitization Inventory Measures Thoughts and Emotions by Sina Ramtin, Marielle Ngoue, David Ring and Teun Teunis in Journal of Patient Experience

sj-docx-3-jpx-10.1177_23743735241273589 - Supplemental material for The Central Sensitization Inventory Measures Thoughts and Emotions Supplemental material, sj-docx-3-jpx-10.1177_23743735241273589 for The Central Sensitization Inventory Measures Thoughts and Emotions by Sina Ramtin, Marielle Ngoue, David Ring and Teun Teunis in Journal of Patient Experience
