# Supplementary material for: The Central Sensitization Inventory Measures Thoughts and Emotions
Source: J Patient Exp. 2024 Aug 14;11:23743735241273589. doi: 10.1177/23743735241273589 (PMC11325304; doi:10.1177/23743735241273589)
Supplement: sj-docx-1-jpx-10.1177_23743735241273589 - Supplemental material for The Central Sensitization Inventory Measures Thoughts and Emotions [file sj-docx-1-jpx-10.1177_23743735241273589.docx]

| Appendix 1: Central Sensitization Inventory Item Groupings in Exploratory Factor Analysis | | | | | |
| --- | --- | --- | --- | --- | --- |
| Questions | Thoughts and feelings | Body Aches | Urinary and visual symptoms | Jaw pain | Uniqueness |
| I feel tired and unrefreshed. | 0.77 |  |  |  | 0.37 |
| My muscles feel stiff and achy. |  | 0.65 |  |  | 0.52 |
| I have anxiety attacks. | 0.33 |  | 0.35 |  | 0.68 |
| I grind or clench my teeth. |  |  |  | 0.49 | 0.76 |
| I have problems with diarrhea and/or constipation. |  |  |  |  | 0.85 |
| I need help in performing my daily activities. |  |  |  |  | 0.84 |
| I am sensitive to bright lights. |  |  | 0.49 |  | 0.68 |
| I get tired very easily when I am physically active. | 0.44 | 0.42 |  |  | 0.55 |
| I feel pain all over my body. |  | 0.72 |  |  | 0.48 |
| I have headaches. |  |  |  |  | 0.88 |
| I feel discomfort in my bladder and/or burning when I urinate. |  |  | 0.57 |  | 0.73 |
| I do not sleep well. |  | 0.46 |  |  | 0.65 |
| I have difficulty concentrating. | 0.60 |  |  |  | 0.48 |
| I have skin problems such as dryness, itchiness or rashes. |  |  | 0.36 |  | 0.76 |
| Stress makes my physical symptoms get worse. | 0.44 |  |  |  | 0.59 |
| I feel sad or depressed. | 0.68 |  |  |  | 0.51 |
| I have low energy. | 0.82 |  |  |  | 0.30 |
| I have muscle tension in my neck and shoulders. |  |  |  | 0.49 | 0.54 |
| I have pain in my jaw. |  |  |  | 0.56 | 0.70 |
| Certain smells, such as perfumes, make me feel dizzy and nauseated. |  |  | 0.34 | 0.32 | 0.74 |
| I have to urinate frequently. |  | 0.35 | 0.55 |  | 0.52 |
| My legs feel uncomfortable and restless when I am trying to go to sleep at night. |  | 0.45 |  |  | 0.69 |
| I have difficulty remembering things. | 0.42 |  | 0.44 |  | 0.58 |
| I suffered trauma as a child. |  |  |  |  | 0.82 |
| I have pain in my pelvic area. |  | 0.31 |  |  | 0.86 |
| All items with a factor loading > 0.3 are shown. |  |  |  |  |  |
