# Supplementary figures and images for: The Central Sensitization Inventory Measures Thoughts and Emotions
Source: J Patient Exp. 2024 Aug 14;11:23743735241273589. doi: 10.1177/23743735241273589 (PMC11325304; doi:10.1177/23743735241273589)

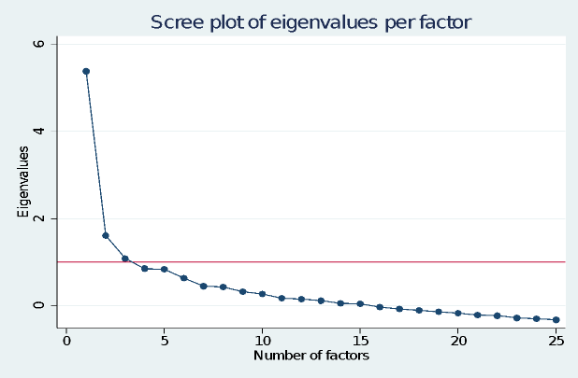

Supplement: sj-docx-2-jpx-10.1177_23743735241273589 - Supplemental material for The Central Sensitization Inventory Measures Thoughts and Emotions [file sj-docx-2-jpx-10.1177_23743735241273589.docx]
