# Supplementary material for: The Central Sensitization Inventory Measures Thoughts and Emotions
Source: J Patient Exp. 2024 Aug 14;11:23743735241273589. doi: 10.1177/23743735241273589 (PMC11325304; doi:10.1177/23743735241273589)
Supplement: sj-docx-3-jpx-10.1177_23743735241273589 - Supplemental material for The Central Sensitization Inventory Measures Thoughts and Emotions [file sj-docx-3-jpx-10.1177_23743735241273589.docx]

| Appendix 3. Fit statistics of the confirmatory factor analysis of Central Sensitization Inventory | |
| --- | --- |
| Fit statistic | Value |
| Variance of items explained (r^2^) | 0.99 |
| Chi-square value (degrees of freedom)^a^ | 374 (178) |
| Likelihood value is equal to degrees of freedom | < 0.001 |
| Root mean squared error of approximation (95% CI)^b^ | 0.084 (0.072-0.096) |
| Comparative fit index^c^ | 0.793 |
| Tucker Lewis index^c^ | 0.756 |
| Standardized root mean squared residual^b^ | 0.074 |
| Coefficient of determination^c^ | 0.994 |
| ^a^If the chi-square value is lower than degrees of freedom, this indicates good model fit. | |
| ^b^Below 0.08 indicates a good fit. |  |
| ^c^A value greater than 0.80 is a better fit. |  |
